# Modeling Raman Spectra in Complex Environments: From Solutions to Surface-Enhanced Raman Scattering

**DOI:** 10.1021/acs.jpclett.4c03591

**Published:** 2025-03-19

**Authors:** Tommaso Giovannini, Sara Gómez, Chiara Cappelli

**Affiliations:** † Department of Physics and INFN, University of Rome Tor Vergata, Via della Ricerca Scientifica 1, 00133 Rome, Italy; ‡ Departamento de Química, Universidad Nacional de Colombia, Av. Cra 30 45-03, 111321 Bogotà, Colombia; ¶ Scuola Normale Superiore, Piazza dei Cavalieri 7, 56126 Pisa, Italy; § IMT School for Advanced Studies Lucca, Piazza San Francesco 19, 55100 Lucca, Italy

## Abstract

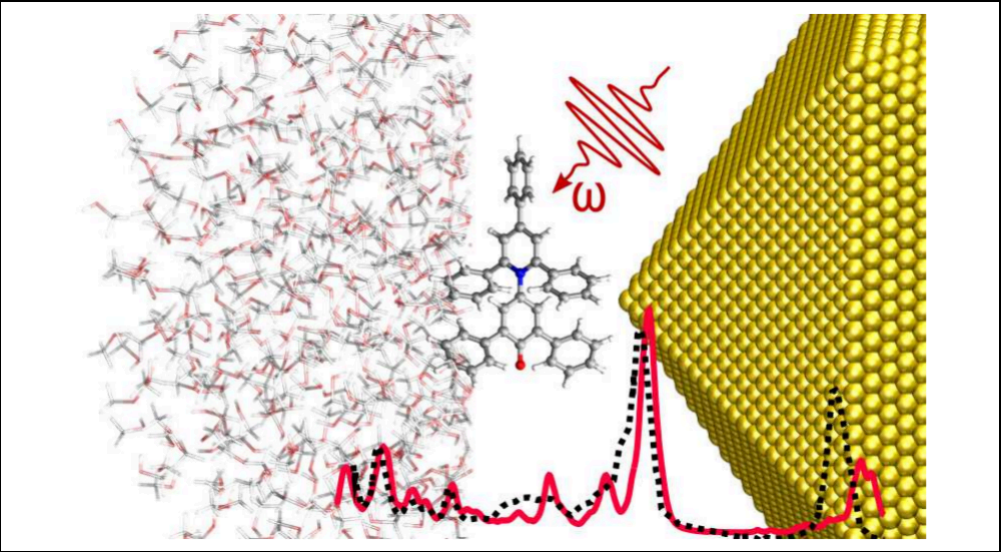

This perspective highlights the essential physicochemical factors required for accurate computational modeling of Raman and Resonance Raman signals in complex environments. It highlights the theoretical challenges for obtaining a balanced quantum mechanical description of the molecular target, integration of target-environment interactions into the Hamiltonian, and explicit treatment of strong interactions such as hydrogen bonding. The dynamical sampling of solute–solvent phase space and the incorporation of plasmonic effects for Surface-Enhanced Raman Scattering (SERS) are also addressed. Through selected applications, we illustrate how these factors influence Raman signals and propose a framework to tackle these challenges effectively, advancing the reliability of theoretical Raman spectroscopy in real-world scenarios.

Raman spectroscopy is a powerful tool for unveiling chemical systems’ structural and electronic properties. It has been employed in numerous applications across several fields,^1−8^ including the real-time monitoring of chemical reactions, examining food products, pharmaceuticals, and chemicals, and is still expanding into many other practical applications. Raman spectroscopy provides in-depth insights into molecular vibrations, which are highly responsive to the nature and strength of chemical bonds. Consequently, it is effective not only for identifying molecules but also for revealing structural details.^9^ Moreover, Raman spectra can indicate changes in a molecule’s environment, making it a useful tool for investigating systems in the condensed phase.^7,8^ Also, Raman is an elective technique to investigate aqueous solutions (the native environment of most biological systems).^7^

Raman is formally a mixed electronic and vibrational spectroscopy, which probes molecular vibrational degrees of freedom by employing an electromagnetic impulse that can be either off- or in-resonance with a molecular electronic transition.^5,10^ In the latter case, the possibility of varying the wavelength of the probing laser permits the unveiling of the effect of the electronic transition on the spectrum.^11−14^ Resonance can be induced by either matching the wavelength of the external probing field with a molecular electronic transition (Resonance Raman - RR),^15−17^ or by stimulating plasmon resonance in the case of plasmonic materials composed of target molecules adsorbed on plasmonic metal (or graphene)-based nanomaterials.^8,18^ In this latter case, the induced plasmon resonance frequency (PRF) hugely enhances the molecular Raman signal, giving rise to Surface-enhanced Raman Spectroscopy (SERS),^8,19−21^ which can also be measured at full resonance regime, in case the PRF is at resonance with an electronic transition of the adsorbed molecule.^22,23^

The complexity of information that is hidden behind Raman spectral patterns, benefits from the coupling between experimental studies and computational simulations.^24^ This is even more necessary in the case of complex systems in the condensed phase,^25−29^ however this kind of modeling is challenging for different reasons. First, the theory level which is employed to describe the molecular system must be accurate enough to capture electron correlation.^16^ In this context, a good compromise between accuracy and computational cost is offered by Time-Dependent Density Functional Theory (TDDFT),^30^ which provides a highly accurate description of Raman activities of most systems.^24^

Second, the effect of the environment (solvent, nanostructured material) must be accurately described. To this end, Raman simulations benefit from the most successful approaches to computational spectroscopy of systems in the condensed phase, i.e. the so-called focused models.^25^ There, the focus is always the target molecule, which is accurately described in all its degrees of freedom with a given quantum mechanical (QM) level, whereas the environment is treated at a lower level of sophistication. This partition aims at accurately reproducing the target-environment interactions, and thus the effect of the environment on the spectrum, rather than the properties of the environment itself.

In most cases, the effects of the environment on Raman spectra can safely be treated classically (QM/classical approach).(28) This greatly reduces the number of degrees of freedom that need to be considered in the QM calculation, which can be limited to those of the target molecule; therefore the dimensionality of the QM problem does not substantially increase compared to the same simulation for the isolated (gas-phase) system. Another substantial advantage of this kind of models is that the presence of the environment is formulated through an interaction term that enters the solute’s QM Hamiltonian.^28^ This permits the use of the machinery of quantum chemistry exactly in the same way as for isolated systems.^31^

The use of a QM/classical approach to model Raman spectra requires a careful definition of the target/environment interactions. Within a classical approximation of the environment, these are generally limited to electrostatic and polarization interactions, which modify the solute electronic structure.^25,32^ Nonelectrostatics (van der Waals) is commonly introduced as a parametrized functional, which affects the nuclear degrees of freedom of the molecular moiety but not its electronic structure.^33^ When specific, strong solute-environment interactions are established, as in the case of solute–solvent hydrogen bonds, an atomistic description of the environment is mandatory to capture the correct physics of the interaction.^25^ This yields an additional computational challenge: a single conformation of the solute–solvent configuration is not a realistic description of the experimental conditions, and the solute–solvent phase space must be carefully sampled employing dynamical approaches, such as molecular dynamics or Monte Carlo.^34,35^ In this way, the dynamical aspects of the environmental effects are automatically taken into consideration in the modeling, providing a physically sound of the experimental conditions. Also, this allows for an effective description of conformational effects arising from structural changes induced by the environment. Finally, to describe SERS, it is crucial to properly describe the plasmonic response of the nanomaterial and its effect on the electronic structure of the molecular system adsorbed on its surface.^36−38^

This perspective focuses on the role of all these aspects in the modeling of Raman spectra, and provides a comprehensive framework for developing a theoretical model capable of accurately incorporating them at a physically sound level. In the following discussion, the theoretical principles and methodologies to calculate the Raman spectrum of a complex system employing a focused approach are discussed. Then, we delve into selected examples that emphasize how the aforementioned factors due to the presence of the environment influence the Raman signal, also illustrating the versatility and applicability of the proposed methods to a wide range of Raman spectroscopy scenarios, including resonance Raman (RR) and surface-enhanced Raman scattering (SERS).

The spontaneous (far-from-resonance) Raman scattering cross-section is usually calculated employing response theory by differentiating the dynamic electric polarizability with respect to normal mode displacements, calculated for a perturbation with angular frequency ω of the light source. Given the vibrational transition polarizability α_*i*_ corresponding to an excitation of the *i*-th normal mode with frequency ω_*i*_, the cross-section σ_*i*_ can be expressed in terms of Raman rotational invariants^39^
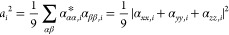
1
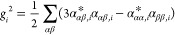
2
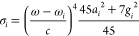
3where the Greek letters indicate *x*, *y*, *z* components and *c* is the speed of light. Most off-resonance Raman calculations rely on the double-harmonic approximation to describe the molecular potential energy surface and expand the vibrational transition polarizability in a Taylor series to first order.^39^ The first derivative term is then calculated analytically or by numerical differentiation with respect to normal coordinates.

The sketched approach assumes the imaginary part of the polarizability to be negligible, which is true when the incident radiation frequency is far from any system’s electronic transition (far-from-resonance). In the case of resonance conditions (RR, SERS, or SERRS) the full sum-overstate expression of the transition polarizability to state *i* needs to be considered^16^
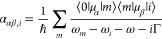
4where μ indicates the electric dipole moment. In eq 4 the summation runs over all vibronic states *m* (with energy ω_*m*_) belonging to the potential energy surface of the resonant electronic state, while Γ is the excited state phenomenological damping constant, representing excited state lifetime.^40^Equation 4 represents the QM definition of electric dipole - electric dipole polarizability α̅, which is generally a complex quantity. Its real part is associated with transmission, while the imaginary component represents absorption. The explicit summation over all vibronic states in eq 4 makes it computationally impractical. Effective polarizability calculations are generally performed using the linear response theory, which can be formulated for exact and approximated wave functions.^31^

For a self-consistent field method, such as Hartree–Fock and Density Functional Theory, the frequency-dependent complex polarizability is computed through the first-order variation of the molecular density under the effect of a time-dependent perturbation. In particular, for a monochromatic uniform electric field **E**^ext^(ω), linearly polarized along the direction α = *x*, *y*, *z*, linear response theory yields to the following coupled-perturbed equations:
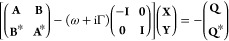
5**A** and **B** matrices for a generic global hybrid DFT functional (defined in terms of the parameters *c*_*x*_, *c*_*l*_) in the molecular orbital (MO) basis (*i*, *j* ∈ occupied; *a*, *b* ∈ virtuals) read
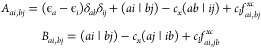
6where ε indicates MO energies, (*rs*|*tu*) two-electron integrals, and *c*_*x*_ and *c*_*l*_ define whether a pure (*c*_*x*_ = 0) or hybrid (*c*_*x*_ ≠ 0) DFT functional is exploited. The right-hand side of eq 5 is written in terms of the perturbation operator which acts on the electronic density *V*^pert^(**r**, ω)
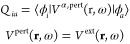
7where *V*^ext^ is the electric potential associated with the external field **E**^ext^.

Once eq 5 is solved for the input frequency ω, the frequency-dependent polarizability tensor α̅_*ab*_(ω; ω′) is obtained as follows^36^
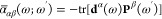
8where **d**^α^(ω) is the dipole matrix of the QM system. The perturbed density **P**^β^ can be computed through the coupled perturbed equations (eq 5), where *X*_*ia*_ = *P*_*ia*_^β^ and *Y*_*ia*_ = *P*_*ai*_^β^.

When the external radiation is not in resonance with electronic excitations, the complex factor in eqs 4 and 5, which takes into account the excited state lifetime, can be neglected. As a consequence, the frequency-dependent polarizability tensor in eq 8 becomes a real quantity and represents the quantum analog of the classical polarizability. By differentiating α̅_*αβ*_(ω; ω′) with respect to the system’s nuclear coordinates, the spontaneous Raman signals can be computed by exploiting eqs 1–3.^41^ The situation becomes more complex when excited states are directly involved, i.e. for Resonance Raman. Attempting to simulate the RR signals using the same methodology employed in the nonresonant case leads to completely erroneous results, as it is easily perceivable by eq 4 (with Γ = 0).^40^ Therefore, to compute RR spectra, the excited state lifetimes must be explicitly considered and the complex coupled-perturbed equations reported in eq 5 need to be solved.^42^ This strategy is particularly effective because it accounts for all the excited states that are potentially in resonance with the external radiation at once. However, from the physicochemical point of view, all excited states are characterized by the same lifetime Γ, which can be a severe approximation.^43^ For the molecular case, some effective alternatives that do not involve solving eq 5 have been proposed. Traditional approaches, such as the transform theory (TT)^10^ and short-time dynamics (STD),^44,45^ have provided valuable frameworks for deriving RR intensities. TT, based on the Kramers–Kronig relationship,^45^ and STD, using a time-dependent redefinition of the Kramers–Heisenberg–Dirac expression,^10^ both rely on approximations that are well-suited for preresonance conditions but often neglect key vibronic interactions. Furthermore, these methods typically assume harmonic potential energy surfaces (PES) and ignore Duschinsky rotations, limiting their applicability for systems with significant vibronic coupling. A more general sum-overstates (SOS) method, based on the harmonic approximation, overcomes these shortcomings by explicitly accounting for both Franck–Condon (FC) and Herberg–Teller (HT) effects and considering differences in the harmonic PES of the ground and resonant states.^40,43,46^ This approach also facilitates the calculation of two-dimensional RR spectra, which offer a richer representation of the scattering process by capturing interference effects from multiple quasi-resonant states.^43^ These methods are defined within the framework of the vibronic theory, and calculate the RR signals either at the time-independent^40,43^ or time-dependent^46−48^ level.

## Raman Spectra in the Framework of Focused Models

**Figure 1:**
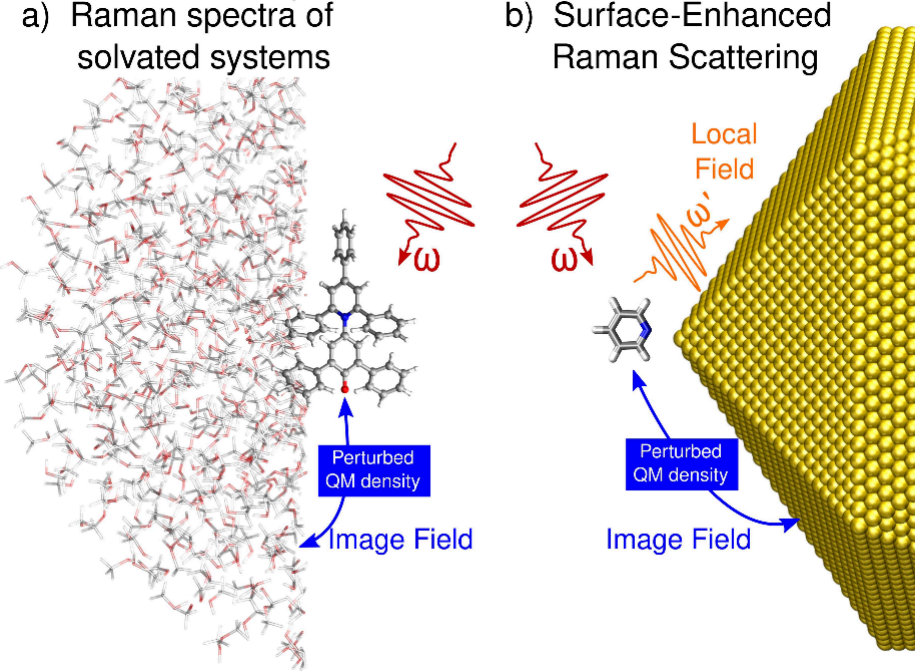
Schematic picture of a molecular system embedded in an external environment under the action of an external radiation and the physical interactions relevant for Raman spectroscopy. (a) Example of off-resonance environment (solution); (b) example of environment at resonance with the external radiation (plasmonic material).

The influence of the external environment on Raman spectroscopy has been widely recognized.^5^ Solvents, biological environments, and plasmonic nanomaterials can significantly affect Raman signals of molecular systems.^8,49,50^ As explained in the introduction, focused models are the most effective methods for modeling Raman spectra of molecular systems interacting with the external environment. The most popular model for computational spectroscopy is the polarizable continuum model (PCM),^51^ which has been extended to the calculation of Raman spectra, both in the off-resonance and resonance regimes and to SERS.^52−56^ PCM effectively describes average environmental effects on molecular signals but lacks any atomistic details, which can be crucial in the modeling of strongly interacting systems (e.g., solute–solvent hydrogen bonding).^28^ These can instead be recovered employing fully atomistic QM/molecular mechanics (MM) approaches,^27^ which offer a more chemically intuitive depiction of the system. Indeed, QM/MM are a valid alternative to continuum descriptions in the calculations of spectral signals, including Raman, RR, and SERS, and are rapidly becoming the golden standard for the simulation of embedded systems of diverse nature.^38,41,50,57^ In the following, such systems are divided into two major classes, based on the physical properties of the environment (see Figure 1). In most cases, Raman spectra of systems in the condensed phase (whether off-resonance or under resonance conditions) are recorded when the surrounding environment is not in resonance with the external exciting radiation. This is the case with solutions and biosystems, which are generally transparent to the external laser radiation (UV–vis Range).^50^ Environments that are at resonance with the external probing radiation are at the basis of SERS enhancement.^8^ In fact, the resonance condition induces surface plasmons on the material (most commonly a rough metal nanostructure based on Ag or Au) that interacts with an adsorbed molecule and enhances its Raman signal of orders of magnitude (up to allowing single-molecule detection).^8^

The two classes of environments differ in their interaction with the external radiation. Consequently, they share the same treatment of the ground state. In QM/MM approaches, the system is partitioned into two parts: the target system, treated at the QM level, and the environment, described fully atomistically at the MM level.^27^ The energy of the total system is thus written as follows
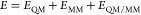
9where *E*_QM_ and *E*_MM_ are the energies of the QM and MM portions, respectively, whereas *E*_QM/MM_ represents the interaction energy between the two moieties. By following the theory of intermolecular interactions, *E*_QM/MM_ is generally decomposed as^27^
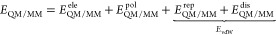
10where *E*_QM/MM_^ele^ is the electrostatic term and *E*_QM/MM_^pol^ is the polarization energy. *E*_QM/MM_^rep^ and *E*_QM/MM_^dis^ indicate Pauli repulsion and dispersion energies, also called van der Waals interactions. The most accurate QM/MM embedding methods, called polarizable embedding, take into account both electrostatics and polarization effects, which are generally written as^27^
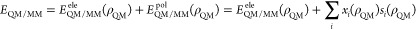
11where *E*_QM/MM_^ele^(ρ_QM_) describes the electrostatic interaction between the QM density and MM electrostatic variables (generally a set of fixed multipoles). The second term describes mutual polarization effects between the QM and MM polarization variables (*x*), which are induced by the QM density through the action of the proper electrostatic operator *s*.^25,35,58^ Remarkably, in most QM/MM methods, van der Waals interactions are treated employing parametrized functions, such as the Lennard-Jones potential, and therefore do not explicitly depend on the QM density, although some notable exceptions have been proposed in the literature.^59,60^

The focus of this perspective stays in the QM/Fluctuating Charges (FQ) family of hybrid, fully polarizable QM/MM approaches, that have been extended in recent years to the calculation of a wide range of spectral properties of systems in complex environments, including plasmonic nanomaterials.^38,61−65^ In QM/FQ and its derivative QM/Fluctuating charges and Fluctuating Dipoles (FQFμ) approach,^32,66^ each environment’s atom is endowed with a charge *q* (and possibly a dipole **μ**) which can “fluctuate”; i.e., they can dynamically respond to the presence of the target and the other MM moieties.^32,66^ In particular, the theoretical foundation of both FQ^67^ and FQFμ is the electronegativity equalization principle,^68^ which says that, at equilibrium, the electronegativities of each atom in a molecule should equalize. The parameters that define the solvent’s response are therefore atomic electronegativities and hardnesses.^67^ FQFμ also features a set of atomic polarizabilities, to specify dipole fluctuation.^32^

For QM/FQ or QM/FQFμ, eq 11 reads^32^
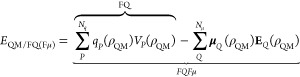
12where *q*(ρ_QM_) and **μ**(ρ_QM_) are fluctuating charges *q* and fluctuating dipoles **μ**, each placed at MM atoms positions, induced by the QM density via its electric potential *V*(ρ_QM_) and field **E**(ρ_QM_), respectively. This means that in eq 11*x* = *q* in FQ, *x* = *q*, **μ** in FQFμ, with *s* being the QM electric potential for *x* = *q*, and the QM electric field for *x* = **μ**. Remarkably, electrostatic interactions are implicitly described by the polarization effects. The QM Hamiltonian is thus modified accordingly to eq 12, i.e.,
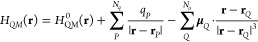
13where *H*_QM_^0^ is the gas-phase QM Hamiltonian. The coupling between eqs 13 and 12 represents the formalization of mutual polarization effects, which are effectively introduced by QM/FQ and QM/FQFμ.

## Environments Off Resonance with the External Radiation: QM/FQFμ

The extension of QM/FQ and QM/FQFμ to Raman spectra implies that the polarizability entering eqs 1-4 is evaluated by including specific contributions arising from the presence of the FQ or FQFμ layers, which modify the QM Hamiltonian as defined in eq 13.^41,64,65,69^ In particular, within an SCF formalism, the coupled perturbed equations (eq 5) need to be modified so that the **A** and **B** matrices explicitly take into account the environmental polarization:^62^
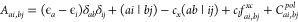
14
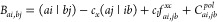
15

**A** and **B** matrices account for an extra term, *C*^*pol*^, which is specified according to the exploited polarizable method, and which accounts for the so-called image field or direct contribution (see Figure 1).^25,26^ For QM/FQFμ, this reads
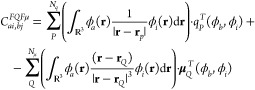
16where, *q*^*T*^ and μ^*T*^ are the perturbed fluctuating charges and dipoles, adjusted to the transition density **P**_*K*_^*T*^ = **X**_*K*_ + **Y**_*K*_.^62,64^ The expression for QM/FQ is obtained from eq 16 by neglecting dipoles’ contribution.

In actual calculations, the molecular ground state geometry is optimized in the presence of the FQ or FQFμ portions, and the electronic density and harmonic PES are modeled by considering the reaction field due to the environment.^25,26^ The calculation of far-from-resonance Raman intensities can then be performed by analytical or numerical differentiation of the dynamic polarizability calculated by solving eq 16. In the case of RR (eq 4), the specific contributions due to the environment depend on the actual approach that is exploited to define RR intensities. In fact, in case a full vibronic model is employed,^43^ the environment’s contributions enter the response equations that are solved to calculate excitation energies and to model the excited state PES and associated normal modes.^40^ As an alternative, RR spectra can be calculated by resorting to the STD approximation.^42^ Intensities are directly computed from the geometrical derivatives of the imaginary frequency-dependent polarizability (eq 4) with respect to normal coordinates,^42,70−72^ with the substantial advantage of avoiding computing excitation energies and properties, which makes calculations afford large molecules and complex environments.^42,73,74^

## Environments at Resonance with the External Radiation: QM/ωFQFμ

QM/ωFQFμ couples a QM Hamiltonian (generally expressed at the DFT level) with the fully atomistic frequency-dependent FQ approach ωFQ and its extension ωFQFμ to treat plasmonic nanostructured materials.^75−77^ By following the general framework of the FQ family of approaches, ωFQ endows the atoms of the nanostructure with a frequency-dependent charge. In the presence of an external monochromatic electric field, charge exchange with nearest neighbor atoms results from Drude conduction^78^ and is modulated by quantum tunneling effects.^75^

ωFQ has successfully been applied to the simulation of the plasmonic response of sodium nanostructures and graphene-based materials.^75,77,79^ However, the underlying Drude conduction mechanism cannot properly reproduce the plasmonic response of *d*-metals, as for instance silver and gold nanoparticles, for which an explicit description of interband transitions in needed.^80−84^ ωFQFμ correctly models such effects.^76^ There, each atom of the plasmonic nanostructure is endowed with both an oscillating charge *q*_*i*_ and an oscillating dipole **μ**_*i*_. The plasmonic response is then assumed to originate from two different mechanisms: the Drude conduction, taken into account by the charges, and the interband transitions, which are modeled in terms of the set of dipoles, which account for the polarizability of the *d*-shell.^76^ Remarkably, these approaches can reproduce the plasmonic response of large nanostructures,^85,86^ bimetallic systems,^87^ defected geometries,^88^ and also nanoparticles below the quantum size limit.^76^

By following the approach reported in ref (89)., SERS spectral intensities can be evaluated through the frequency-dependent complex polarizability tensor in eq 4, suitably introduced in eqs 1. However, to describe SERS, it is crucial to account for the electric field generated by the plasmonic substrate under the action of the external field (local field, see Figure 1).^38^ To this end, the perturbation operator which acts on the electronic density defined in eq 7 is modified as follows^38^
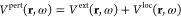
17where *V*^loc^ is the local field operator.^38^ Therefore, two additional contributions to the TDKS equations arise for QM/ωFQ and QM/ωFQFμ: the local field in the right-hand side, and the so-called image field (direct contribution) to the left-hand side matrices (*C*^*QM*/ωFQ(Fμ)^ – see eq 16),^38^ which determines the response of the classical variables to the perturbed density.^90,91^ On the other hand, the explicit contribution to the right-hand side is associated with the surface plasmon resonance and is responsible for the electromagnetic enhancement mechanism, i.e. the enhancement of the Raman signal due to the enhanced electromagnetic field in the surface proximity.^8^ We remark that neglecting the latter contribution yields unphysical results, and the final computed spectrum does not take into account the physics of the SERS phenomenon.

Once eq 5 is solved for the input frequency ω, the frequency-dependent polarizability tensor α̅_*αβ*_(ω; ω′) is obtained as in eq 8, where **d**^α^(ω) also accounts for the local field operator along the direction α, i.e.,^38^
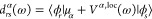
18From the physical point of view, the presence of the local field operator in eq 18 can be explained by the fact that the total scattered field from the molecule-nanostructures composite system contains two contributions: the scattered field from the molecule and the reflected field, which is generated by the molecule and reflected on the plasmonic nanostructure (see also Figure 1).^89,92^

## Normal Mode Definition in Focused Models

An important point that needs to be addressed when computing Raman spectra is how to define the system’s normal coordinates. In fact, once molecular polarizabilities are defined, for either nonabsorbing or absorbing external media, they need to be differentiated with respect to the system’s normal coordinates to get Raman activities. The definition of normal modes in a complex system is not trivial, because the target’s and the environment’s motion cannot be decomposed uniquely. This decomposition can be performed at increasing levels of sophistication. Note, however, that in a focused model, normal modes are computed for the target system only, while neglecting the vibrations of the environment. This is consistent with the Partial Hessian Vibrational Approach (PHVA).^93−95^ Such an approximation can be generally performed at different levels of accuracy. In the roughest approximation, which we call A0, for a given structure of the complex system (e.g., a structure extracted from an experimental database, MD trajectories etc.) normal modes are computed without optimizing the target system.^74^ In this way, the target conformations are preserved, however it will not generally lie in its energy minimum. Although this approximation might be rather crude, it can give an initial insight into the vibrational spectral shape if the actual target geometry is not too different from the equilibrium one.

As an alternative, normal modes for each representative target structure are obtained by limiting geometry optimization to the target and by keeping the environment frozen. This approach is properly defined within the PHVA. The target’s vibrational degrees of freedom are separated from those of the environment. Notably, this method, which is the most used for vibrational calculations at the QM/MM level, preserves the environment’s fluctuations in the case of structures extracted from MD simulations.

With increasing sophistication, the target’s normal modes can be computed on a reference structure (e.g., the isolated molecule or within a continuum description of the environment). The reference and “distorted” structures can be related to each other by employing a fitting (superimposing) procedure that uses rotations and translations to minimize the root-mean-squared deviation (RMSD) between the two sets of coordinates. For each structure, a 3 × 3 transformation matrix, A1 is defined, which provides the best alignment between the target optimized structure and its geometry in the current complex structure. The transformation matrix is applied to the normal modes of the reference-optimized target structure to project them onto the actual complex structures.^74^

As a final approximation, the adiabatic-molecular dynamics generalized vertical Hessian Ad-MD|gVH approach, A2,^96^ can be exploited. For each structure, the reduced-dimensionality Hessian resulting from projecting out the soft coordinates from the ground state Hessian is considered. This procedure yields a new set of frequencies and normal modes, over which Raman activities can be obtained by numerical differentiation. The use of one method or another may lead to significant differences in the final spectra, as will be shown in the following discussion.

## Illustrative Applications

**Figure 2:**
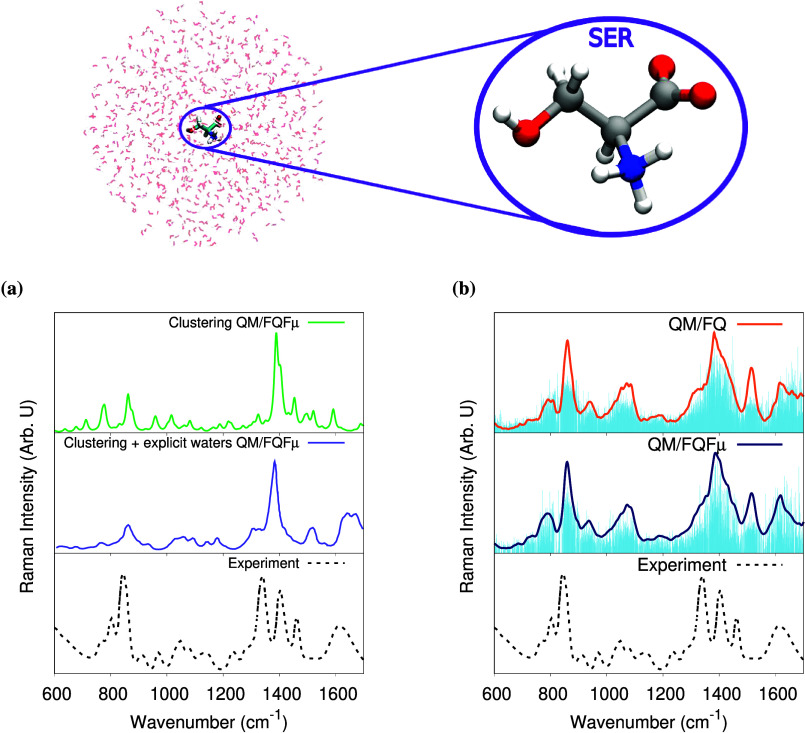
Computed Raman spectra of SER in aqueous solution. (a) QM/FQFμ Raman spectra as obtained with the clustering approach and with conformers from clustering plus explicit water molecules in the QM region. (b) QM/FQ and QM/FQFμ Raman spectra from averaging 400 snapshots extracted from an MD trajectory. All QM/MM spectra were computed at the B3LYP/TZP level for the QM portion. Sticks (blue vertical lines) were convoluted with a Lorentzian line shape, using a full width at half-maximum (fwhm) equal to 6 cm^–1^. Computational data taken from ref (98). The experimental Raman spectrum, reproduced with permission from ref (99). Copyright 1993 John Wiley and Sons is also reported.

To discuss different aspects that need to be considered for constructing a successful model to model Raman spectra of complex systems, we resort to a set of selected applications. First, we focus on the physicochemical aspects that affect the simulation of Raman signals of molecular systems in solution and interacting with biological matrices. The theoretical and computational background is mature enough to accurately consider both the conformational and configurational degrees of freedom and strong solute–solvent interactions, such as hydrogen bonding. This is possible through the definition of a suitable computational protocol that can account for such effects at the same level. The protocol integrates the effects of the environment on Raman signals, as defined by the specific contributions to polarizabilities, with the accounting of the specific spatial arrangement of the classical environment around the QM target system (see refs (25), (91), and (97) for more details). The first step involves (*i*) the definition of the system, demarcating the QM and classical portions. Then, (*ii*) a conformational and configurational sampling is performed, by resorting to strategies such as MD simulations, which may also imply a specific reparametrization of existing force-fields. Once the phase space is explored, the computational sample is prepared (*iii*) by extracting a set of representative structures. In the case of Raman calculations in solution, such structures are cut in spherical droplets, of a typical radius of less than 20 Å (see Figure 2, top panel). Just a few hundred of them are needed to give excellent computed results.^50^ (*iv*) QM/classical Raman calculations are carried out on the frames obtained at the previous step, at a given QM computational level. Different sets of parameters for the classical portion may be exploited. Finally, (*v*) individual results are extracted, analyzed, and averaged to produce final spectra. The convergence of the spectra when varying the number of configurations is assessed (and in case, the number of snapshots/calculations is increased) and a final comparison with experimental data is made. Further refinement of some of the above stages could be needed, then the protocol may be repeated if necessary.

Finally, we move toward the simulation of SERS signals of molecules interacting with plasmonic substrates, which substantially increases the complexity of the phenomenon.

## Conformational and Configurational Sampling

The effect of conformational and configurational sampling is evident when investigating the Raman spectra of Serine (SER) in an aqueous solution.^98^ SER (zoomed in the top panel of Figure 2) is a simple amino acid with an −OH group as a substituent that opens a lot of possibilities to interact with solvent molecules, in addition to the carboxylate and the protonated amino group in the zwitterionic form. It is worth mentioning that due to the strong contacts of SER with water molecules via hydrogen bonding, the use of a continuum solvation approach to simulate the Raman spectra of this system would provide an incomplete picture of the solvation phenomena.

To obtain a rigorous sampling of the solute–solvent phase space and explore the SER’s conformations, 30 ns-long MD simulations were employed. Three ways to extract structures for computing spectra were explored: ***clustering***, ***clustering + explicit water molecules***, and ***taking a set of 400 snapshots*** from the MD trajectories. In the former, five conformers were identified for SER using the GROMOS clustering tool, and in the second, the water molecules belonging to the first solvation shell as extracted from the first minima in the RDFs were explicitly included in the QM portion of the five dominant configurations. In the latter, about 720 solvent molecules were incorporated into the calculations and treated with the FQ force field. The resulting Raman spectra are compared in Figure 2.

When analyzing the spectrum obtained from the weighted average on the five representative structures to the experimental one reproduced from ref (99), Figure 2, panel (a), it is clear that the main differences entail the position of the peaks, their relative intensities, and the inhomogeneous band broadening, which mostly arises from the many solvent configurations sampled during the MD, and is missing in the clustering approach. Notice also that a complete quantum treatment of the closest water molecules to possibly take into account the different orientations of important solvent molecules does not solve the issue of the relative intensities of the peaks. In contrast, as shown in Figure 2, panel (b), QM/FQ and QM/FQFμ spectra computed as an average of over 400 snapshots extracted from the MD are in very good agreement with the experimental data. The main discrepancy is reported in the region 1300–1500 cm^–1^, where the experimental spectrum features three intense peaks, while two bands are barely predicted by QM/FQ and QM/FQFμ, probably because two peaks are fused into a broad band. The overall very good agreement with the experiments emphasizes the need to consider not only the solute conformations but also the solvent distribution around the solute extensively, which leads to the spreading of the blue sticks and ultimately to the emergence of a natural broadening. The same behavior has also been reported for systems containing small amides,^100^ dipeptides,^50^ and drugs in aqueous solution^57,101^ and the effect of the conformational and configurational sampling is more exacerbated when the solutes have specific contacts with the solvent molecules. Therefore, although clustering methodologies reduce the computational cost associated with QM/MM calculations on a larger set of configurations, they have serious flaws and are therefore not recommended for the prediction or reproduction of Raman spectral signals. For chiroptical spectroscopic, such as Raman optical activity (ROA), where sign patterns are significant, the situation could be even worse.^98^ Finally, it is worth remarking that, for SER in aqueous solution, QM/FQ and QM/FQFμ provide similar spectra (see Figure 2b). In fact, only minor discrepancies in the relative intensities (e.g., at 1500 cm^–1^) are reported, highlighting that, in this case, the effect of dipoles (i.e., QM/FQFμ) is negligible.

## Hydrogen Bonding, Refinement of the Embedding, and Effect of the External Frequency

**Figure 3:**
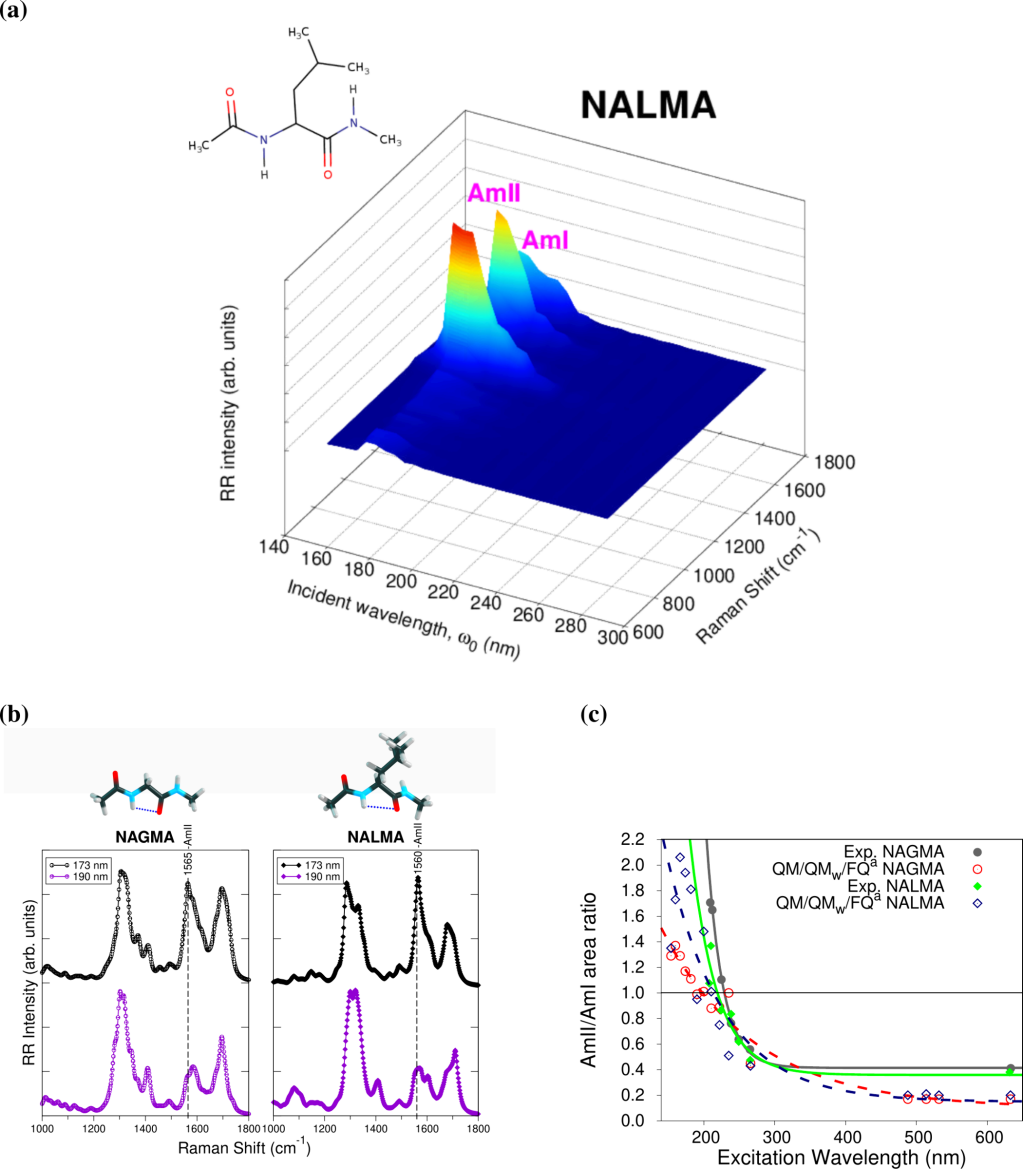
(a) RR intensity dependence on the exciting frequency used to irradiate NALMA in aqueous solution. (b) Comparison between the QM/QM_*w*_/FQ UVRR spectra simulated at 173 and 190 nm for NAGMA and NALMA dipeptides. (c) Experimental and computational scanning of the ratio of the AmII/AmI areas along the excitation wavelength. RR intensities were calculated with the vibronic approach at the B3LYP/6-311++G(*d*, *p*)/FQ level of theory, using 15 TD-DFT excited states in each case and a damping factor of 200 cm^–1^. RR spectra were broadened using Lorentzian functions with an fwhm of 20 cm^–1^. Data taken from ref (50).

The capability of vibrational spectroscopy methods like (Resonance) Raman as valuable tools for refined structural analysis of peptides and proteins in aqueous solution has been revealed in the study of the protein prototypes NAGMA and NALMA (see Figure 3b).^50^ These solutes were experimentally and theoretically investigated by combining cutting-edge synchrotron-based spectroscopic measurements^102−105^ and highly accurate multiscale simulations. The latter exploited the fully polarizable QM/FQ approach coupled to MD simulations, which correctly describe the physicochemical aspects of the solute–solvent interactions, and is useful to provide explanations to experimental findings such as the *selective enhancement* of the amide II (AmII) mode^15,106^ observed at the shortest excitation wavelengths for both dipeptides in aqueous solution and not for their microcrystalline form.^50^

Two sets of FQ parameters, FQ^*a*^ from ref (67) and FQ^*b*^ from ref (107), were used to model the Raman spectra of NAGMA and NALMA. This change constitutes an initial way of refining the embedding since enabling the FQ^*b*^ parameters makes it possible a more accurately account for the electrostatics and polarization effects in the QM Hamiltonian. Indeed, using the FQ^*b*^ parametrization moved the RR spectra of the dipeptides toward a better reproduction of the experimental measurements.^50^

The influence of the external frequency on the Raman spectra of NAGMA and NALMA was examined in terms of resonance Raman excitation profiles (RREP), consisting of the variation of the RR intensities when changing the incident wavelength. The QM/FQ modeled RREP of NALMA is shown in Figure 3(a). Notably, strong UVRR intensities for AmI, AmII, and AmIII (vibrations having significant contributions from the C–N stretching mode) are to be expected when properly tuning excitation wavelengths toward ∼180 nm (190 nm is the experimental report) that is the excitation associated with the π → π* transition. The orbitals involved in this transition have large contributions from the C–N regions of NAGMA and NALMA and not only belong to the solutes but also involve the closest water molecules, thus suggesting a charge transfer between the peptides and the solvent.

To understand the preferential enhancement of AII when approaching the maxima in the absorption spectra, a few solvent molecules were included in the QM portion of the system, in what is called the QM/QM_*w*_/FQ approach, further refining the embedding. QM/QM_*w*_/FQ RR spectra are displayed in Figure 3(b) for two selected wavelengths, namely, 173 and 190 nm, that exemplify the selective enhancement of AmII over AmI. An explanation at the molecular level for the origin of the selective enhancement of amide signals is that the presence of the water molecules helps concentrate the π orbitals in the C–N regions and shortens the C–N distances, which ultimately leads to intensifying the UVRR vibrations that have large components of the C–N stretching, in particular the AmII signal.^50^ The experimentally and computationally estimated ratio of the areas of amide modes AmII/AmI as a function of the excitation wavelength is shown in Figure 3(c). In the comparison with experimental data, it should be pointed out that in the simulations the incident frequency, ω_0_ (or excitation wavelength) is chosen to be different from the calculated vertical energy by the same amount as the experimental incident wavelength varies with respect to the measured absorption maximum.^40^ The unparalleled agreement between theory and experiment is reached only when the solvent molecules are linked to the C=O and N–H groups of the dipeptides, demonstrating the critical role of hydrogen bonding in determining amide spectral features in the resonance Raman spectroscopy. Hence, modeling specific hydrogen bond interactions could be required in case of particular hydration dynamics around solutes.

## Increasing Complexity in Raman: The Treatment of Biological Matrices

The previous examples dealt with the Raman signals of systems in solution where the QM/MM partitioning was effortless because no covalent boundary exists between the solute and the solvent.^25^ Also, the size of the solutes was small enough to handle all vibrations with the PHVA, and there were FQ (and FQFμ) parameters to treat the solvent. However, the situation can be way more complicated when it comes to chromophores embedded in biological matrices.

**Figure 4:**
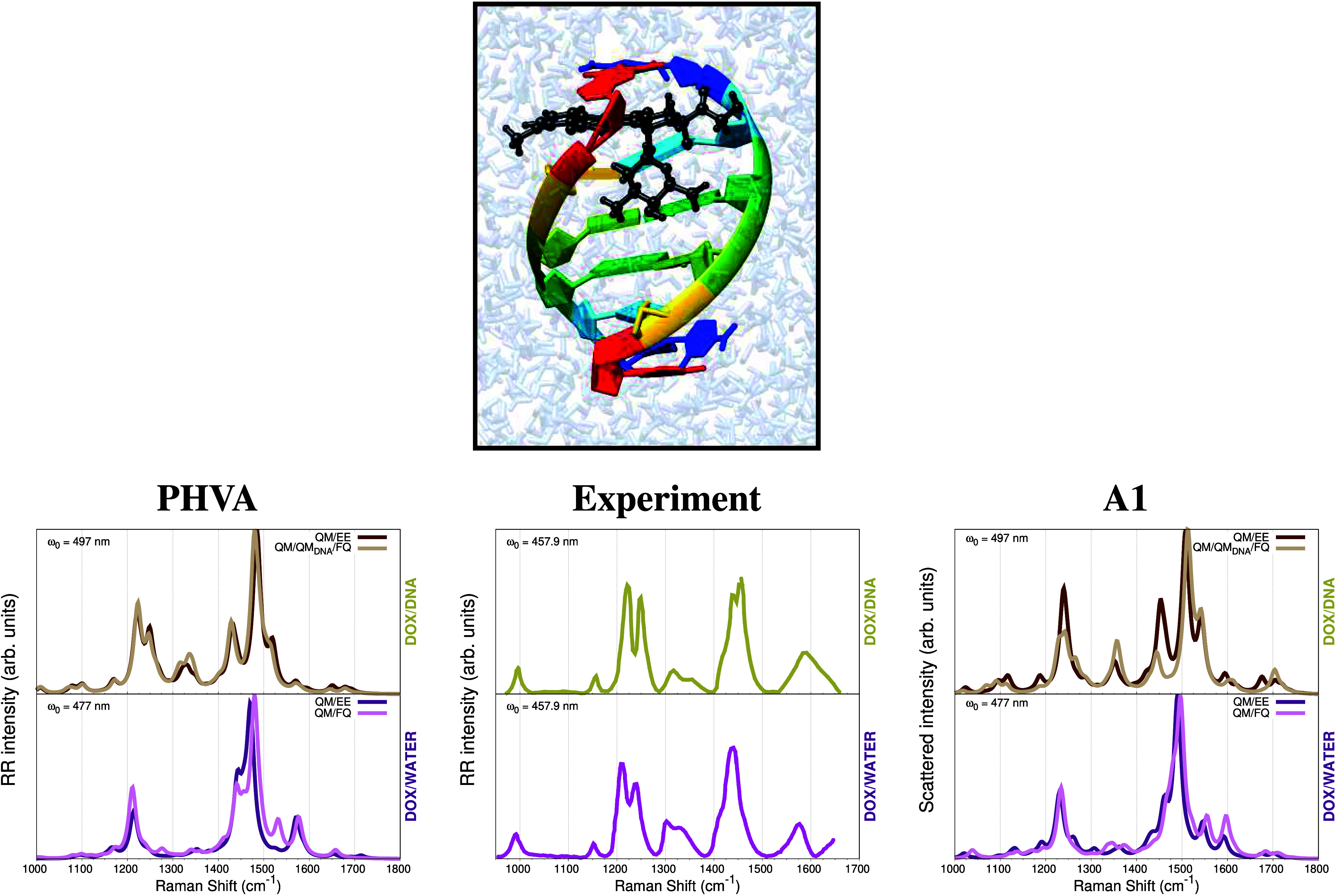
Top panel: Example of a configuration where DOX is intercalated into a DNA sequence. Bottom panel: Experimental^112^ and computed RR spectra of DOX-water and DOX-DNA-water resulting from applying the PHVA and the A1 approaches to compute normal modes. Computational data are taken from ref (74). RR intensities were calculated through complex polarizability derivatives using a damping factor of 500 cm^–1^. A Lorentzian shape with a fwhm = 20 cm^–1^ is used in the broadening. QM level: B3LYP/DZP. All spectra are scaled such that the maximum intensity is unity.

By its selectivity and sensitivity advantages, many biological systems are experimentally studied using the RR technique.^108,109^ Notwithstanding, the computational simulation of such combined spectroscopy is far from trivial because it requires assembling a model where electronic transitions, normal modes, and polarizabilities are consistently integrated.^74^ Doxorubicin (DOX), a routinely used chemotherapy agent,^110,111^ intercalated into DNA is an interesting system with available RR experimental data.^112−114^ A configuration of that system, borrowed from the MD simulations performed in refs (115) and (116) is shown in Figure 4. Just to mention the challenges associated with the calculation of the normal modes, treating systems such as solvated DOX or a DOX complexed with DNA implies including hundreds (201) of vibrations. In addition, and as discussed above, the configurational phase space of the target-environment system must be extensively sampled to get reliable spectroscopic signals. This means that the vibrational analysis has to be performed on multiple configurations until arriving at a converged spectrum. Another key point to consider is the type of embedding to be employed, because due to the lack of FQ parameters for the nitrogenous bases, the DNA and the water molecules could be represented either by fixed charges in an electrostatic embedding fashion (QM/EE),^117,118^ or by separating them and treating the DNA with a QM’ level while all water molecules as FQs (QM/QM_DNA_/FQ). DOX is noncovalently bound to the DNA sequence facilitating the split in regions,^119^ but for covalently bound ligands, making cuttings and inserting link atoms might be needed.

After sampling the configurational phase-space through MD simulations, the four different levels of sophistication to compute normal modes (PHVA, A0, A1, A2, see above) have been tested for the described DOX-containing systems.^74^ The result of modeling RR in DOX-Water and DOX-DNA-water using PHVA and A1 is presented in Figure 4 along with the experimental spectra reported by Angeloni et al.^112^ Both computational approaches allow us to easily identify the main features of the RR spectra in aqueous and DNA solutions. In the PHVA, the DOX moiety is optimized on each snapshot while freezing the rest of the nuclear coordinates. PHVA-derived spectra in Figure 4, bottom-left panel, agree with the experimental ones, with a good reproduction of the experimental frequencies but with several differences in the relative intensities of the peaks. In contrast, such discrepancies are reduced when the A1 scheme is employed, as seen in Figure 4, bottom-right panel. The A1 strategy is computationally cheaper than PHVA because it does not need to optimize the chromophore on each snapshot, but to apply a rotation matrix to travel between the geometry of DOX in any of its lowest energy conformations to the actual geometry of DOX on each snapshot, thus benefiting from previously computed normal modes.^74^ The slightly better agreement with the experiments found for spectra acquired with the A1 scheme vs those of the PHVA, confirms the reliability of the recently proposed method. Therefore, the extended atomistic multiscale computational protocol and the A1 methodology are very versatile and thus suitable for promising Raman applications in complex biosystems.

## Environment at Resonance with the External Radiation: SERS

**Figure 5:**
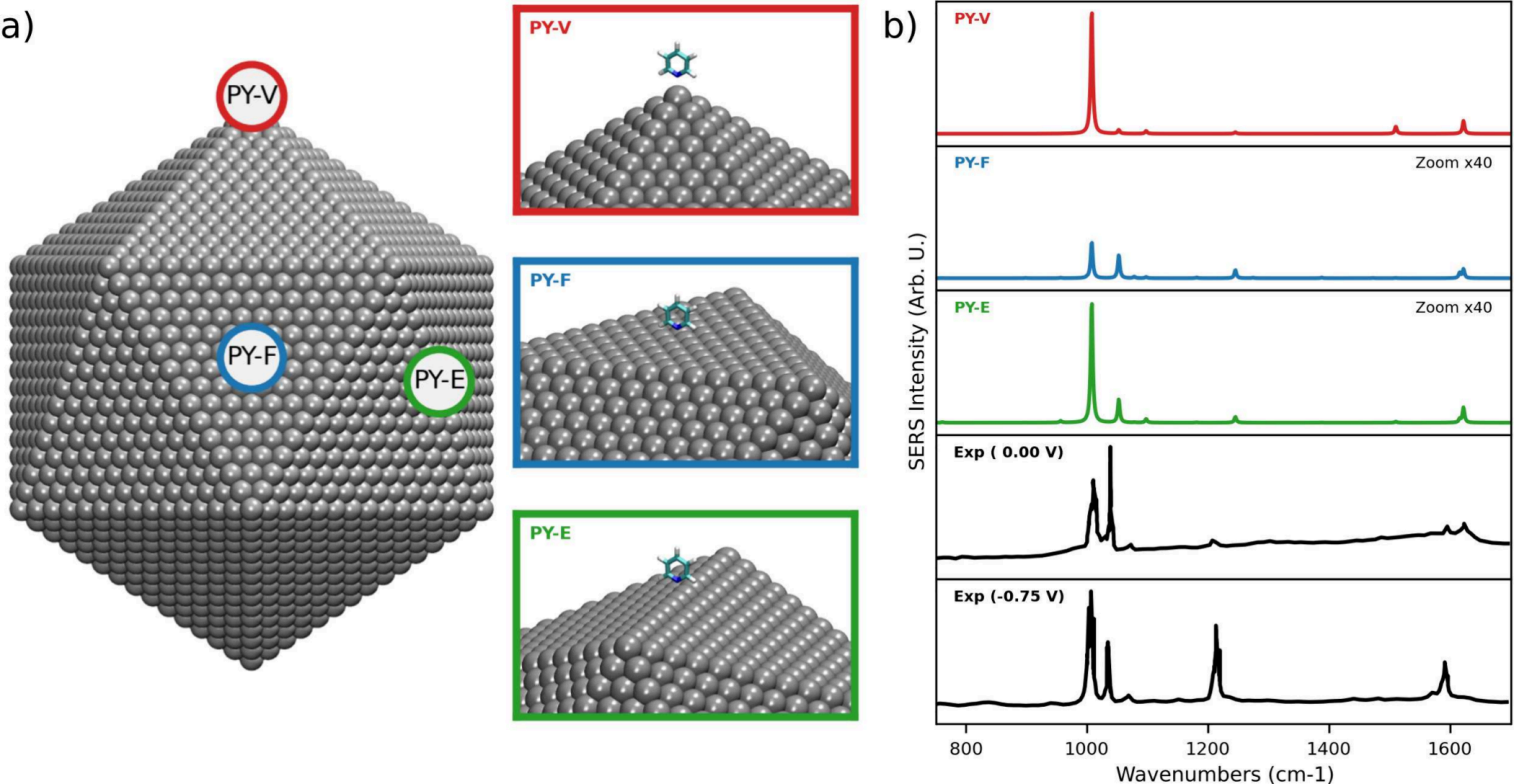
(a) Graphical depiction of the studied configurations of pyridine adsorbed on Ag Ih NP (vertex – PY-V – face – PY-F – edge – PY-E); (b) QM/ωFQFμ SERS spectra of PY adsorbed on Ag Ih NP as a function of the adsorption site, together with the experimental spectra^120^ at 0.0 eV and −0.75 eV external bias. The SERS signal is computed at the PRF of the NPs (3.51 eV). Adapted from ref (38). Copyright 2023 the authors, published by American Chemical Society.

When a molecule interacts with a plasmonic substrate, the complexity of the overall system substantially increases. Beyond the previously discussed physicochemical aspects that affect the liquid phase, the absorbing properties of the nanostructure must be accurately described to model surface-enhanced Raman scattering (SERS) signals. Remarkably, exploiting the same approach used for modeling Raman signals in nonresonant environments is inconsistent with the real physics of the system and leads to incorrect results. Physically grounded modeling must therefore account for the effect of the plasmon field on molecular signals, as this interaction represents the main physical mechanism for signal enhancement (commonly referred to as electromagnetic enhancement – EM).^8^ In this section, we illustrate a representative example of fully atomistic QM/classical modeling of SERS spectra, using pyridine (PY) adsorbed on a large Icosahedral (Ih) Ag NP (10179 atoms) as a case study.^38^ In this case, a minimal modeling of the system was employed: PY was treated at the QM level, the Ag NP was modeled fully atomistically using the ωFQFμ approach, and the interactions between the two entities and the external radiation were fully considered. Given the complexity of the system, the study has been limited to three specific PY-NP configurations, which have been selected as representative points of the Ih morphology (see Figure 5a): the vertex (PY-V, red), edge (PY-E, green), and face (PY-F, blue). In all cases, PY is adsorbed perpendicularly to the NP surface at a fixed distance of 3 Å, with the nitrogen atom closest to the NP. The resulting QM/ωFQFμ SERS spectra calculated at the plasmon resonance frequency (PRF) of the selected NP (3.51 eV) are shown in Figure 5b.

As a result of the PY-NP interaction, the SERS spectrum exhibits significant variations. This is particularly evident in the relative intensities of the two strongest Raman peaks, corresponding to the ring breathing mode (1008 cm^–1^) and the symmetric bending of the α-H (1053 cm^–1^). For all selected adsorption sites, the relative intensity of the bending mode (1053 cm^–1^) decreases compared to the ring breathing mode (1008 cm^–1^), almost vanishing for the PY-V configuration (red). The relative PY-NP arrangement affects not only the spectral profile but also the enhancement factors. In fact, the enhancement factor for PY-V is almost 2 orders of magnitude higher than those computed for both PY-F and PY-E (note the highlighted scaling factors in Figure 5b). This is consistent with the tip effect,^78^ generally reported by all plasmonic materials. In the PY-V configuration, PY is adsorbed on the sharpest region of the Ih NP, leading to a significantly stronger localized electric field, and therefore, a larger enhancement as compared to the other arrangements. The observed differences in spectral shape and enhancement factors between the structures can be attributed to the inhomogeneity of the electric field induced by the NP geometry, which varies substantially from the vertex (PY-V) to the face (PY-F) of an Ih morphology.

QM/ωFQFμ calculations can also be compared with experimental data taken from the literature (Figure 5b).^120^ Notably, QM/ωFQFμ results are based on an ideal representation of the experimental conditions. Instead, measured spectra result from the interplay between several effects that are not necessarily taken into account in the modeling, such as solvent effects, the coating of the metal electrode, external bias applied, impurities, and the roughness of the metal surface. For these reasons, similar to previous computational studies,^89^ the comparison between computed and experiments may only be qualitative.

For PY adsorbed on Ag electrodes, experimental SERS spectra have been measured at various bias potentials (see Figure 5).^120^ At zero bias (0.00 V), the experimental spectrum is dominated by two prominent bands at approximately 1000 and 1050 cm^–1^, with their relative intensities inverted at higher voltages (see bottom panels). Remarkably, these two bands also appear as the most intense features in the computed QM/ωFQFμ SERS spectra across all investigated PY–PS configurations. The experimental spectra are also characterized by a band at approximately 1200 cm^–1^, which becomes more pronounced as the external bias increases (see bottom panels in Figure 5b). Such a band is often attributed to a charge transfer (CT) mechanism, which is not accounted for by QM/ωFQFμ, which as stated above, only describes the EM mechanism. In fact, the 1200 cm^–1^ band is nearly absent in the SERS spectrum of the most intense configuration (PY-V, top panel), further supporting the experimentally driven hypothesis that this band arises from CT effects.

## Future Challenges

The accurate and efficient modeling of Raman and SERS remains an active and evolving area of research. While significant progress has been made, especially in liquid-phase systems, numerous challenges persist. Addressing these challenges will require advances in computational methodologies and the integration of advanced techniques to bridge the gap between theory and experiments.

In the context of liquid-phase systems, the current fully atomistic models have reached a high level of maturity, being able to effectively account for specific solute–solvent interactions as well as the intricacies of the solute–solvent phase space. However, as common to all fully atomistic models, the computational protocols that are generally exploited for computational spectroscopy require a series of delicate steps, which must be carefully performed to guarantee the outcome. For vibrational spectroscopies, such as Raman and RR, this is even more evident because a critical step is the normal-mode analysis, which relies on computationally expensive geometry optimizations, especially for large and flexible molecules. For this reason, we see as particularly promising the development of computational techniques that can bypass the explicit optimization of molecular geometries by using approximate methods to estimate vibrational normal modes. Some attempts in this direction have been recently made relying on gradient projection.^96^ However, to guarantee vast applicability, it is mandatory to create workflows that are as automated and “black box” as possible, allowing nonexpert users to perform high-quality simulations without deep intervention in technical details. This would make computational Raman spectroscopy more accessible across various disciplines. Another aspect that substantially affects the quality of liquid-phase simulation is the sampling. Capturing dynamic effects, such as anharmonicity and coupling to the environment, may require enhanced sampling techniques in MD. Advanced methods, including metadynamics and umbrella sampling,^34,121^ could provide a more accurate representation of vibrational spectra by sampling rare events and long-time scale dynamics effectively, especially for highly flexible molecular systems.

The modeling of SERS is still at the dawn due to the inherent complexity of the systems involved. As mentioned above, the experimental SERS setups involve the interplay of multiple factors, such as solvent effects, metal coatings, external bias, impurities, and surface roughness, which can significantly influence the spectra. It is thus crucial to properly translate the computational protocols designed to model the liquid phase to such systems, properly including the physical differences between the two setups. This demands the development of advanced force fields able to capture the interactions at the nanoscale for sampling the phase space.^122,123^ This goes in parallel with the development of scalable and cost-effective methods to describe the possible chemical bonding between adsorbates and nanostructures. Accounting for these factors will help capture chemical enhancement effects, generally related to Charge-Transfer excitations,^8^ which are crucial for a comprehensive modeling and understanding of SERS mechanisms.

Beyond these specific areas, there are emerging opportunities to push the boundaries of Raman modeling. Among them, Hyper-Raman, which probes higher-order polarizability tensors, offers complementary insights to traditional Raman.^124,125^ However, its modeling requires theoretical developments to describe nonlinear optical effects accurately, particularly in complex environments.^126^ From a different perspective, time-resolved Raman spectroscopy provides a dynamic view of molecular and plasmonic processes on ultrafast time scales.^127−131^ Incorporating real-time simulations into Raman modeling frameworks will require innovations in nonequilibrium QM methods and faster dynamics simulations to align with experimental time resolution. These techniques in fact introduce additional complexities,^132−137^ as the calculation of time-resolved spectra requires accounting for evolving excited-state potential energy surfaces and transient normal modes. Differently from steady-state Raman, where vibrational modes are computed for a well-defined electronic state, time-resolved approaches necessitate tracking these modes dynamically across different excited-state manifolds. Future advancements in theoretical frameworks should aim to bridge this gap, enabling a more accurate computational description of ultrafast Raman spectroscopies, especially in complex systems.

Tackling emerging challenges in hyper-Raman and time-resolved spectroscopy will open new avenues for the theoretical exploration of molecular and plasmonic systems. These advancements will bridge the gap between computational predictions and experimental observables, driving progress across chemistry, physics, and materials science.
